# New Insights in Central Venous Disorders. The Role of Transvenous Lead Extractions

**DOI:** 10.3389/fcvm.2022.783576

**Published:** 2022-02-23

**Authors:** Giulia Domenichini, Mathieu Le Bloa, Patrice Carroz, Denis Graf, Claudia Herrera-Siklody, Cheryl Teres, Alessandra Pia Porretta, Patrizio Pascale, Etienne Pruvot

**Affiliations:** Cardiology Service, University Hospital of Lausanne, Lausanne, Switzerland

**Keywords:** transvenous lead extractions, leadless cardiac pacemaker, subcutaneous cardioverter defibrillator, venous stenosis, superior vena cava syndrome

## Abstract

Over the last decades, the implementation of new technology in cardiac pacemakers and defibrillators as well as the increasing life expectancy have been associated with a higher incidence of transvenous lead complications over time. Variable degrees of venous stenosis at the level of the subclavian vein, the innominate trunk and the superior vena cava are reported in up to 50% of implanted patients. Importantly, the number of implanted leads seems to be the main risk factor for such complications. Extraction of abandoned or dysfunctional leads is a potential solution to overcome venous stenosis in case of device upgrades requiring additional leads, but also, in addition to venous angioplasty and stenting, to reduce symptoms related to the venous stenosis itself, i.e., the superior vena cava syndrome. This review explores the role of transvenous lead extraction procedures as therapeutical option in case of central venous disorders related to transvenous cardiac leads. We also describe the different extraction techniques available and other clinical indications for lead extractions such as lead infections. Finally, we discuss the alternative therapeutic options for cardiac stimulation or defibrillation in case of chronic venous occlusions that preclude the implant of conventional transvenous cardiac devices.

## Introduction

Cardiac implantable electronic devices (CIED) are the first line treatment for a large spectrum of cardiac arrhythmias, consequently the implanting rates has constantly increased over the last decades due to the aging population and expanding indications (1–4).

Transvenous systems currently remain the most common CIED, while leadless pacemakers and subcutaneous defibrillators (S-ICD) still represent only a minority amongst CIEDs. Importantly, the lead component of CIEDs represents the “Achilles' heel” of transvenous devices. Moreover, the implementation of new technology in CIEDs as well as the increasing life expectancy have been associated with a higher incidence of transvenous lead complications over time, including malfunction, venous stenosis and lead-induced tricuspid regurgitation (1, 2, 5). Stenosis of the subclavian vein, the innominate trunk and/or the superior vena cava have been reported in up to 50% of implanted patients (6, 7). Lead extraction, in addition to venous angioplasty and stenting, represents an appealing approach to overcome venous stenosis in case of device upgrades or to treat venous stenosis-related symptoms (i.e., superior vena cava (SVC) syndrome) (1, 2).

This review provides an overview about the role of transvenous lead extraction procedures in the treatment of central venous disorders related to transvenous leads such as venous stenosis and occlusion. Furthermore, the different extraction techniques currently available and the other clinical indications for lead extractions are described. The alternative options for cardiac stimulation or defibrillation in case of chronic venous occlusions precluding the conventional CIED implantation are also explored.

## Central Venous Stenosis Related to Transvenous Leads

### Epidemiology

The incidence of venous stenosis following transvenous lead implant documented by contrast venogram is ranging from 25 to 64% (6–11); this variability is mainly related to the degree of stenosis adopted as inclusion criteria among the different series. In a study by Morani et al. (11), severe stenosis (>75%) have been found in 27% of patients referred for CIED revision after a median time from first implantation of 66.7 ± 46.4 months. Total venous occlusions are also relatively common and have been reported in 6% of patients 6 months after pacemaker (PM) implantation (8) and in up to 26% of patients requiring CIED revision 6.2 years after the first implant (6).

Total venous occlusions are more frequently located at the level of the brachiocephalic vein (7), whereas the most common site of stenosis is the subclavian vein followed by the brachiocephalic trunk, even though both subclavian and brachiocephalic vein are often involved together (11).

### Risk Factors and Pathophysiology

Patients' demographics, implant techniques and lead characteristics have been analyzed by several studies to assess the risk for venous stenosis after CIED implantation. While prolonged implantation time (>60 min), perioperative complications, previous use of temporary PM and left ventricular ejection fraction (LVEF) ≤ 40% are associated with an increased risk of venous stenosis or occlusion (8, 10, 12), the role of the number of implanted leads and lead characteristics including lead type (PM and implantable cardiac defibrillator (ICD) leads), diameter and insulation (silicone, polyurethane and Optim™) still remains controversial (6, 10, 11, 13–18). Interestingly, Haija et al. (6) found that not only the number of implanted leads, but also the total lead diameter as the sum of all implanted lead diameters, is a risk factor for venous stenosis or occlusion. Furthermore, they described an association between multiple lead implant procedures and venous stenosis, as confirmed also by Morani et al. and Czajkowski et al. (11, 12) corroborating the hypothesis of the endothelium trauma as “primum movens” of a cascade where the inflammatory reaction may result in venous stenosis promoting development of granulation tissue development and fibrous capsule formation around the transvenous lead (8, 10). This hypothesis supports the role of anticoagulant therapy in reducing the risk of venous thrombosis after CIED implant (16, 17), but not the risk of venous stenosis itself (13).

### Clinical Presentations

Venous stenoses are often asymptomatic because of venous collateral formation ensuring venous drainage (7, 8, 13, 14, 16, 17) so that they are usually discovered accidentally at the time of CIED revision. The extension of the collateral circulation increases proportionally with the degree of venous stenosis (7) and the development of collateral superficial veins across the clavicle has been shown to be a sensitive marker of severe venous obstruction (9).

SVC syndrome is the manifestation of severe obstruction or occlusion of the SVC and has been documented in 0.1–3.3% of patients implanted with transvenous leads (19, 20). Typical signs and symptoms consist of facial and neck oedema, non-pulsatile distended neck and chest veins, dyspnea and cough, arm oedema and dizziness (19, 21). The severity of clinical presentation depends on the level of obstruction (upper SVC proximal to the azygos vein, azygos vein, and distal to the azygos vein), onset of obstruction and establishment of venous collaterals (19). After transvenous lead implant, symptoms of SVC syndrome might occur months to years later, as documented in a meta-analysis by Riley et al. (22) where the median time between PM implantation and development of SVC syndrome was 48 months (range several hours to 396 months).

### Therapeutic Options

**Figure 1:**
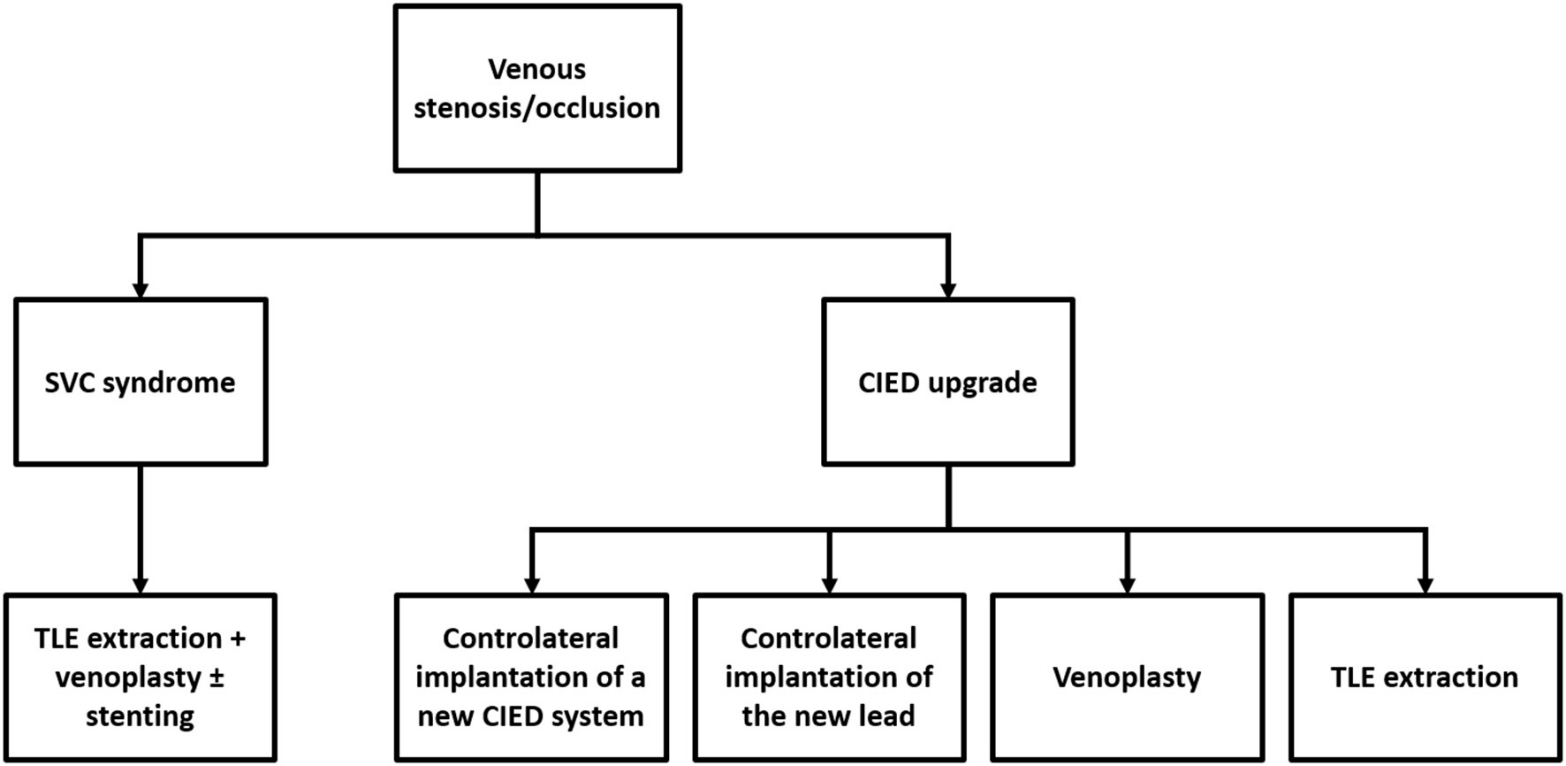
Current therapeutic approach to treat or overcome venous stenosis/occlusion related to transvenous cardiac leads.

Chronic venous stenoses related to transvenous leads usually do not need specific treatments unless in case of SVC syndrome or CIED upgrade requiring new transvenous lead implant. Figure 1 illustrates the current therapeutic approach to treat or to overcome venous stenosis in these cases.

#### SVC Syndrome

Transvenous lead extraction (TLE) followed by venoplasty ± stenting at the SVC level represents the first-choice approach (19, 23–25). Historical treatments like anticoagulation and/or thrombolysis, surgical interventions (i.e., SVC bypass using a spiral saphenous vein conduit; reconstruction of the SVC using a pericardial patch; thrombectomy) and venoplasty alone have been abandoned due to the high risk of recurrences (22). Stenting without lead removal has also been performed, but this approach potentially exposes the patient to lead failure and theoretically precludes the feasibility of percutaneous lead extraction procedure such as for lead infection.

#### CIED Upgrades

Controlateral implantation of a new CIED system, controlateral implantation of the new lead with subcutaneous tunnelisation to the old pocket, venoplasty and TLE extraction of abandoned leads allow overcoming venous stenosis in these cases (11, 26–29). No data comparing procedural and long-term results of these different approaches are available so far, hence the decision to adopt one technique rather than the other should be driven by a risk-benefit assessment case by case. Specific considerations should also be made as abandoned leads contra-indicate magnetic resonance imaging (MRI) and current recommendations discourage the implant of more than 4 leads through the SVC because of increasing risk of occlusion (2, 26). The feasibility and the safety of venoplasty at the time of CIED upgrades have been recently reported in a large series by Worley et al. (27). A total of 373 consecutive venoplasties were performed over 11 years for central and/or peripheral obstruction with a success rate of 99%. No existing leads were damaged, and there were no complications related to venoplasty during the procedure or before discharge. However, the feasibility of this approach remains strictly dependent on the experience of the performing physician and the volume of the center.

## Transvenous Lead Extraction as Therapeutic Option in Case of Venous Stenosis Related to Transvenous Leads

### General Indications for Lead Extraction

Currently the adopted definition of CIED lead extraction is “any lead removal procedure in which at least one lead requires the assistance of equipment not typically required during implantation or at least one lead was implanted for longer than 1 year” (1, 2). CIED infections (including pocket infections with or without bacteriemia, CIED endocarditis and occult bacteriemia with suspected CIED infection) represent the main indication for CIED extractions amounting to 46–56.9% in the largest series on TLE procedures (ELECTRa, LExICon and PROMET study) (30–32). The second most frequent reason for lead extraction is lead dysfunction [38.1% of cases in the ELECTRa study (30)] followed by abandoned leads. In this case, as for lead dysfunction, the rational for extraction would be to reduce the intravascular lead burden especially in young patients (1, 33). Other indications for lead extraction are: lead complications such as SVC syndrome; venous stenosis, preventing new lead implantation; access to MRI in case of abandoned or dysfunctional lead; chronic pain due to periosteal reaction at the lead insertion site (1). An emerging indication for lead extraction is severe tricuspid regurgitation caused by a lead interfering with tricuspid valve leaflet mobility or coaptation in absence of right ventricular or tricuspid valve annulus dilatation and damage of tricuspid valve leaflets (5, 34, 35). However, given the absence of data on large series, this approach should be reserved to very selected patients.

### Lead Extraction Techniques

Percutaneous techniques represent the first line approach for TLEs. Open surgical extractions are associated to an increased risk of major complications and mortality compared to percutaneous extractions (36, 37), therefore they are currently indicated for patients with systemic infection and large lead vegetations (>20 mm) (38). However, also in these cases, a percutaneous approach associated to the aspiration of the lead vegetations has been recently proposed and preliminary results seem encouraging (39, 40).

**Figure 2:**
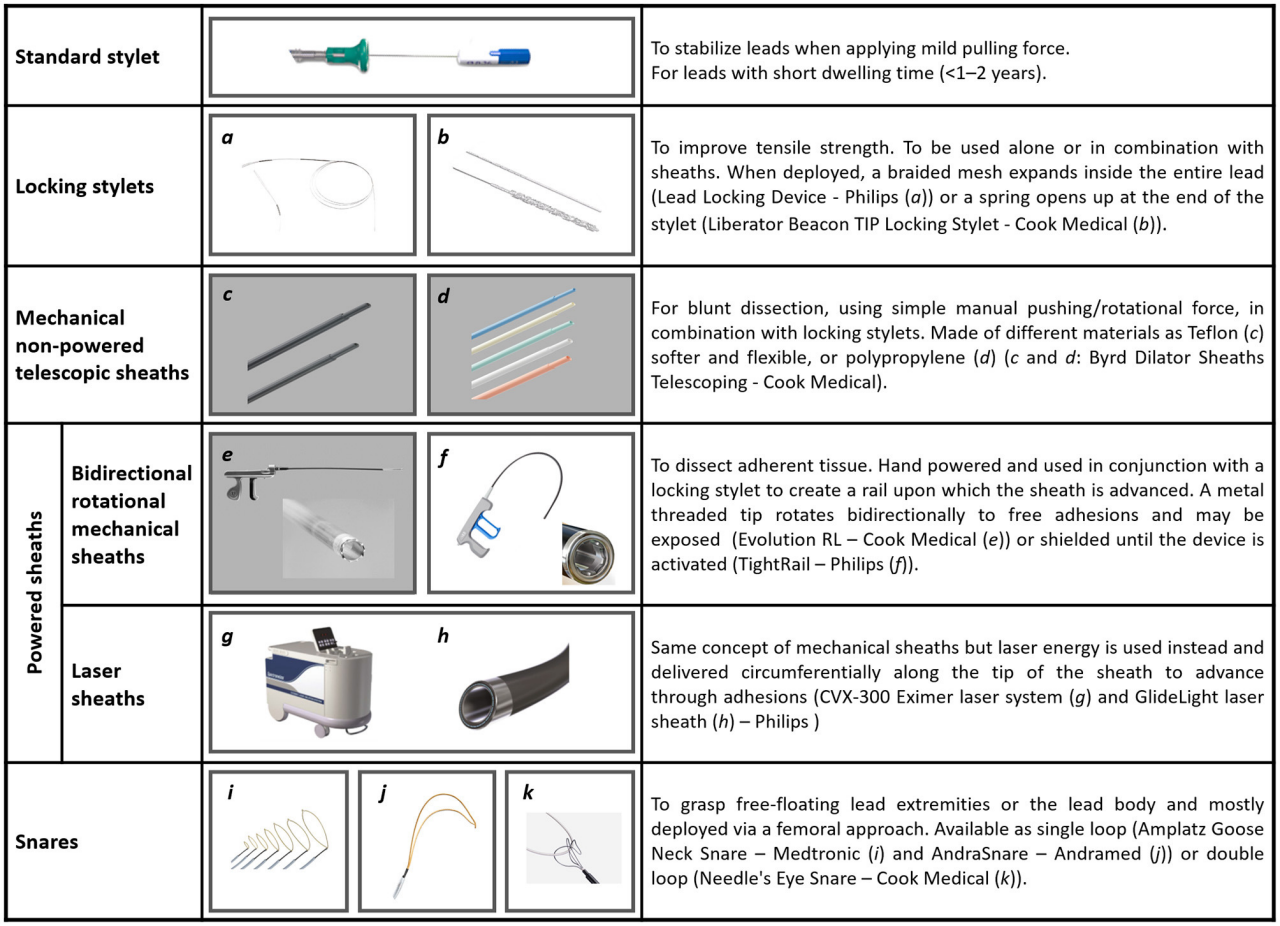
Tools and techniques currently in use to perform percutaneous TLEs [Figure text adapted from Bongiorni et al. (1) and from (41)].

**Figure 3:**
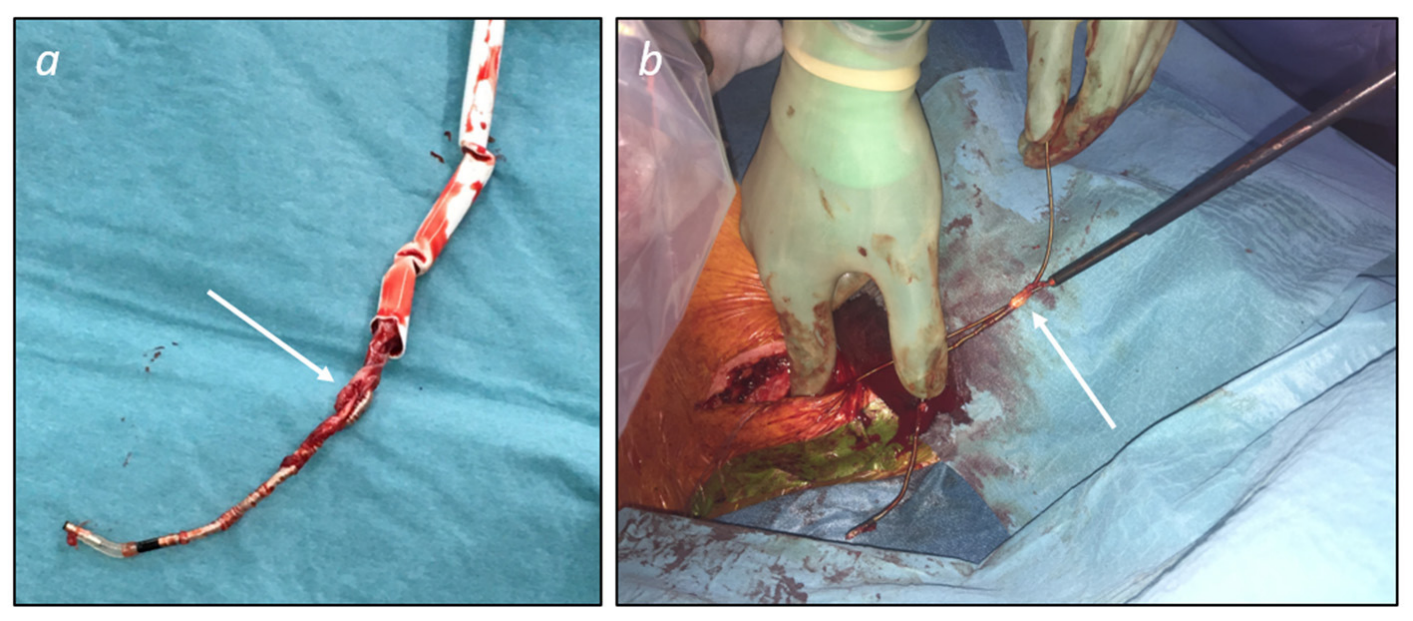
Examples of transvenous leads extracted using mechanical sheaths. **(a)** Ventricular pacing lead (passive fixation) extracted using a manual non-powered sheath (Philips SightRail™ 11.5F). Tissue adhesions (arrow) at the tricuspid valve level were dissected advancing the sheath while applying a pushing rotational force that explains kicking of the shaft. **(b)** Active fixation pacing leads extracted while advancing a rotational mechanical sheath (Philips TightRail™ 9F) mounted on the atrial lead. Once the retroclavicular adhesions were overcome, the atrial lead was easily extracted and dragged out together with the ventricular lead because of fibrous tissue (arrow) bounding the leads together at the brachiocephalic vein level.

Tools and techniques for percutaneous TLEs are illustrated in Figure 2. Additional tools (not shown in Figure 2) are grasping devices, like myocardial biopsy forceps or endoscopic graspers, mostly employed to retrieve lead fragments and occlusion balloons able to control bleeding in case of vascular tear. Figure 3 shows two examples of transvenous leads extracted using mechanical sheaths in our center. Usually, the percutaneous TLEs are performed by using the same venous access as the one of the original implant procedure, even though a femoral or a jugular access may be used as alternative or in case of bailout procedure (1). Furthermore, a “stepwise” approach is normally adopted so that the operator moves from simple (e.g., gentle traction using a locking stylet) to more complex strategies and tools (e.g., powered extraction sheaths) during the procedure according to the success of each single step (1). A pre-procedural contrast venography is also helpful to identify regions of venous stenosis/occlusion and adhesion sites (1, 2, 42).

### General Outcome of Percutaneous TLEs

Percutaneous TLEs have been shown to be safe and effective in several large series of extraction procedures. In the ELECTRa study (30) 3,510 patients underwent TLEs in 73 centers across Europe between 2012 and 2014. Complete removal of the target leads was obtained in 95.7% of cases, whereas the clinical success rate (defined as the achievement of the clinical outcome for which the TLE was scheduled without the occurrence of major complications) was 96.7%. Procedure-related major complications, including death, occurred in 1.7% of patients. The more frequent procedure related complications were cardiovascular requiring pericardiocentesis, chest drainage and/or surgical repair in 1.4% of patients. Powered and non-powered sheaths were used in the majority of the procedures (63.6%) with laser sheaths accounting for 19.3%. In LExICon study (31) all TLE procedures were performed using laser methods only. The procedural and clinical results were similar to the ELECTRa study with a procedure success rate of 96.5% and a clinical success rate of 97.7%. Procedural complications occurred in 1.4% of patients, including 4 deaths (0.28%). More recently the PROMET study (32) showed similar efficacy and safety of laser methods when using mechanical and rotational sheaths with a procedural success rate of 96.5%, major complication rate of 1% and peri-operative or procedure-related mortality rate of 0.18%.

### Percutaneous TLEs in the Context of Central Venous Disorders: Techniques and Results

As previously discussed, TLE has been proposed as a part of therapeutical approach in case of venous disorder related to transvenous leads.

Retrospective data from relatively small series and case reports (23–25) documented the safety and the feasibility of TLE as a part of percutaneous management of the SVC syndrome, even though symptom recurrences may occur several months or even years later, requiring additional venoplasties mostly because of intrastent stenosis. Extraction techniques, procedural results and clinical outcomes of the largest studies available in the literature on TLEs in patients with SVC syndrome are reported in Table 1.

**Table 1:** Data from the largest series available in literature on TLEs as a part of percutaneous management of SVC syndrome.

**Reference**	**Treatment strategy**	**Nb of pts**	**TLE technique**	**Nb of leads**	**TLE success rate**	**TLE major** **complications**	**N. of CIED** **reimplantations**	**F-up duration**	**Clinical outcome**
Riley et al. (22)	TLE + SVC stenting	6	Manual traction (2 pts) Laser (4 pts)	13 [mean dwelling time 71 mo (4–253)]	92%	None	5 (83%) (all transvenous CIEDs)	48 mo (10–100)	Symptom recurrences in 3 pts requiring venoplasties because of intra-stent stenosis
Fu et al. (23)	TLE + SVC venoplasty	13	Laser (10 pts) Mechanical sheaths (2 pts) Laser + mechanical sheaths (1 pt)	25 [mean dwelling time 107.3 mo (0.2–213.3)]	100%	None	9 (69%) (8 transvenous CIEDs, 1 S-ICD)	12 mo	No symptom recurrence
Arora et al. (24)	TLE + SVC venoplasty (+ SVC stenting in 5 pts)	16	Manual traction (3 pts) Mechanical sheath and/or Laser (13 pts)	37 [mean dwelling time 5.8 yrs (2–12)]	96.6%	1 SVC tear requiring surgery	11 (68%) (5 transvenous and 5 epicardial CIEDs, 1 S-ICD)	5.5 yrs (IQR 2.0–8.5)	Symptom recurrences in 4 pts requiring venoplasty and stenting in 1 case

TLE of abandoned leads has also been confirmed to be a safe and effective solution to overcome venous stenosis at the time of CIED revision or upgrade. Furthermore, the presence of venous obstruction itself seems to have no impact on procedural major complications (42). In the series from Barakat et al. (28) 503 patients underwent abandoned lead extraction because of lead dysfunction, lead recall or venous stenosis (37 patients). Powered sheaths were used in 75% of the TLEs and the overall success rate was 96.6%. Major complications occurred in 1% of patients and damage to pre-existing leads related to the extraction procedure was documented in 3.8% of cases. Sohal et al. (29) evaluated 71 patients who underwent lead extraction because of venous occlusion preventing CIED revision. All leads (129 in total, mean dwelling time 80 ± 62 months) were completely extracted using laser sheaths, and the new leads were successfully implanted across the obstruction in 94% of cases. There were two major complications consisting in infection of previously sterile sites but no peri-procedural mortality. Post-procedural device checks were satisfactory in 92% of cases with a mean follow-up of 26 ± 19 months. However, these data (28, 29) reflect the experience of high-volume centers and suggest that the adoption of these strategies should be limited to experienced operators only. Finally, post-extraction venous occlusion and embolization of collateral veins have been described following TLE procedures using laser sheaths, because of vessel injuries promoting thrombus formation (43). The impact of this phenomenon is particularly relevant in case of TLEs performed for symptomatic venous obstructions and should be taken into account when defining the TLE strategy. Anticoagulation therapy seems to prevent post-operative thrombosis but more evidence are required to support its use in this specific context.

## Alternative Therapeutic Options for Cardiac Pacing or Defibrillator in Case of Chronic Venous Occlusion

**Figure 4:**
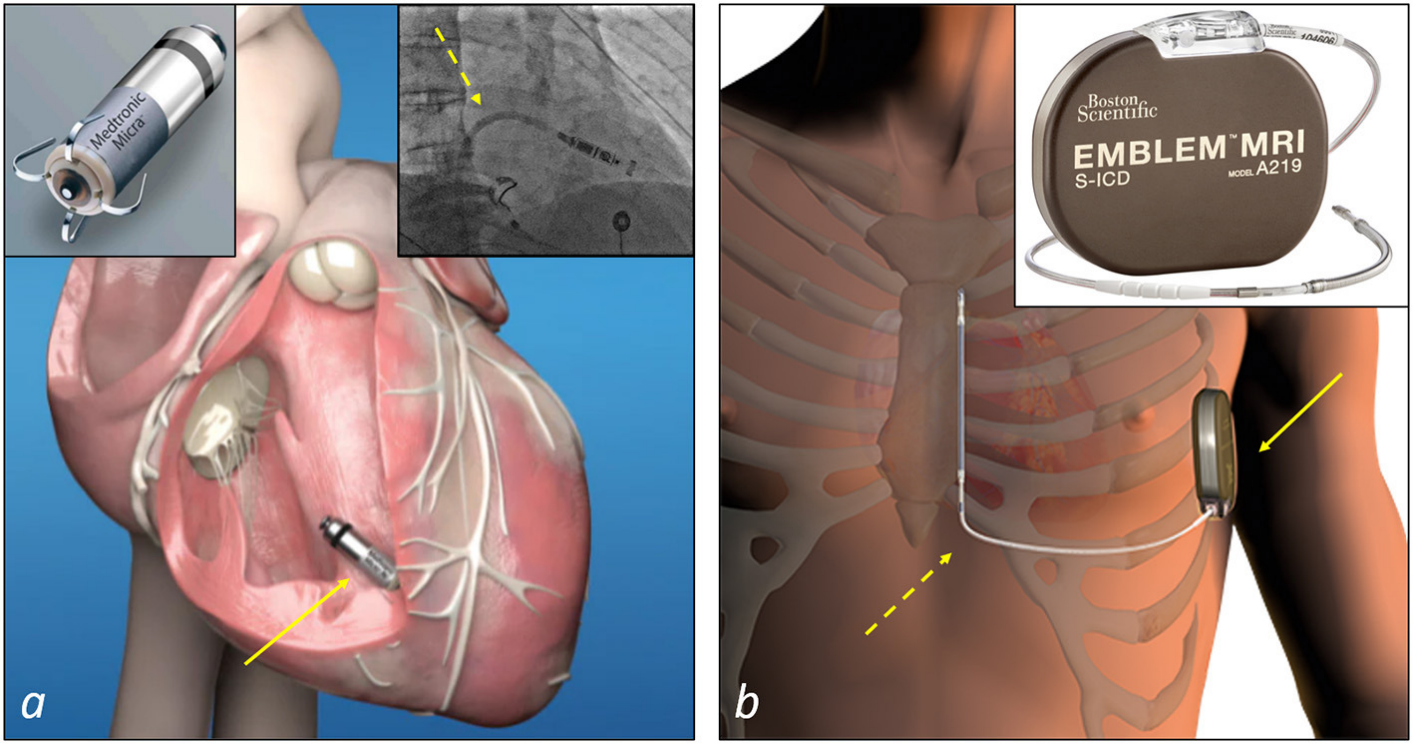
Examples of non-transvenous CIEDs systems to deliver cardiac pacing or defibrillation therapy. **(a)** A leadless PM (Micra™ Medtronic, top-left corner) and the site of implant at the apical-septal level of the right ventricle (arrow). The insert at the top right corner shows a step of a Micra™ implantation: the catheter delivery system (dashed arrow) is advanced into the right ventricle to deliver the device. **(b)** A subcutaneous ICD (Emblem™ MRI Model A219, Boston Scientific, top-right corner) and the site of implant of the device (solid arrow) and the lead (dashed arrow). The device is implanted in the left axillary region in an intermuscular pocket created between the serratus anterior and the latissimus dorsi muscles, and connected to the lead implanted in the subcutaneous tissue of the parasternal region of the chest.

Surgical epicardial leads and intracardiac leadless PMs represent the recommended therapeutic alternatives mostly used to deliver cardiac pacing in case of no venous access or occluded veins at the upper extremities preventing the implantation of transvenous leads (44). In a recent survey of the European Heart Rhythm Association on the use of leadless PMs in Europe, an anticipated difficult vascular access has been reported as the main reason of choice of these devices compared to conventional transvenous PMs (45). Compared to an epicardial system, the leadless PM implantation procedure is relatively less invasive as these devices are implanted percutaneously into the right ventricle using customized catheter-based delivery systems through the femoral vein (44) (Figure 4a). Nevertheless, leadless pacemakers have limited pacing modalities and memories, so that the decision to implant these devices also depends on the clinical indication for cardiac pacing.

In patients who meet the indication for an ICD and do not need pacing for bradycardia, ventricular tachycardia termination or cardiac resynchronisation therapy, subcutaneous ICD (S-ICD) is the recommended therapeutic option in case of inadequate vascular access (46, 47) (Figure 4b). However, in general, not all potential candidates to an S-ICD are eligible because of inadequate sensing of the QRS and/or T waves of the ECG, with under and oversensing of these waves that can prevent or result in the delivery of inappropriate shocks. To limit this problem, an ECG screening is performed prior to the implantation. Therefore, in these specific cases and when a pacing treatment is required, a surgical approach remains the only solution in patients with vascular access issues.

## Conclusions

Central venous disorders related to transvenous leads are a relatively common finding in CIED population because of an increasing life expectancy and expanding CIED indications leading to multiple CIED box changes and revisions. Several approaches have been proposed over the last years to treat symptoms related to venous occlusion (e.g., SVC syndrome) or to overcome venous stenosis in the context of CIED upgrade. TLEs is a rather safe and feasible solution in these specific cases, but needs to be performed by experienced operators in high volume centers with a surgical back-up. Nevertheless, new CIED technologies, such as leadless PM or subcutaneous ICD, have recently become available and currently represent the recommended alternative to conventional transvenous CIEDs in selected patients with central venous disorders. Furthermore, S-ICDs are especially recommended in young patients requiring cardiac defibrillation therapy to preserve the venous capital over decades.

## Author Contributions

GD and EP contributed to the conception and designed of the manuscript. GD wrote the draft of the manuscript. All authors contributed to manuscript revision, read, and approved the submitted version.

## Conflict of Interest

The authors declare that the research was conducted in the absence of any commercial or financial relationships that could be construed as a potential conflict of interest.

## Publisher's Note

All claims expressed in this article are solely those of the authors and do not necessarily represent those of their affiliated organizations, or those of the publisher, the editors and the reviewers. Any product that may be evaluated in this article, or claim that may be made by its manufacturer, is not guaranteed or endorsed by the publisher.
